# Supplementary material for: Integrative taxonomy of Haemaphysalis (Acari: Ixodidae) from the Western Ghats, India: Morphological and molecular characterization and implications
Source: PLoS One. 2026 May 7;21(5):e0348592. doi: 10.1371/journal.pone.0348592 (PMC13152140; doi:10.1371/journal.pone.0348592)
Supplement: S1 Table — Newly generated sequences are marked with asterisk. (DOCX) [file pone.0348592.s001.docx]

**S1 Table.** Specimens used for molecular analysis with their provenances, BOLD information and GenBank accession numbers. Newly generated sequences are marked with asterisk.

| **Species** | **Provenance** | **Voucher code** | **BOLD:Process id** | **BOLD:BIN** | **GenBank Accession no.** |
| --- | --- | --- | --- | --- | --- |
| *Haemaphysalis* sp*.* | Australia | Isolate Tick 7-1 | GBA27476-15 | BOLD:ACV7002 | KM821502 |
| *Haemaphysalis* sp*.* | India | S2N3 |  |  | PQ585789* |
| *Haemaphysalis* sp*.* | India | S2N4 |  |  | PQ585790* |
| *Haemaphysalis* sp*.* | India | Isolate TK-3 | GACO5054-19 | BOLD:ADV1020 | MH937512 |
| *Haemaphysalis aculeata* | India | P6N1 |  |  | PQ410419* |
| *Haemaphysalis aculeata* | India | S6AM |  |  | PQ410420* |
| *Haemaphysalis adleri* | Israel | 916a |  |  | PV612054 |
| *Haemaphysalis bancrofti* | Australia | Isolate C9b2 | GACO4802-19 | BOLD:ACV7002 | MH043268 |
| *Haemaphysalis bispinosa* | India | Isolate NIVEDI_2019_PK30 | GACO5389-19 | BOLD:ADV1020 | MK863389 |
| *Haemaphysalis bispinosa* | India | Isolate NIVEDI_2019_PK38 | GACO5425-19 | BOLD:AEA8995 | MN106409 |
| *Haemaphysalis bispinosa* | Bangladesh | TK0R2 |  |  | MK269314 |
| *Haemaphysalis bispinosa* | Malaysia | UPMBTU T07 |  |  | OR742954 |
| *Haemaphysalis bispinosa* | India | K3N2 |  |  | PQ432748* |
| *Haemaphysalis bispinosa* | India | S3N1 |  |  | PQ432749* |
| *Haemaphysalis bispinosa* | Vietnam | Isolate VQ9 |  |  | PQ439198 |
| *Haemaphysalis bispinosa* | Indonesia | Isolate CDMusuk |  |  | ON778586 |
| *Haemaphysalis bispinosa* | India | Isolate Ollur |  |  | PV746300 |
| *Haemaphysalis campanulata* | China | Isolate A39 |  |  | OM368277 |
| *Haemaphysalis concinna* | China |  |  |  | NC034785 |
| *Haemaphysalis cornigera* | China | Isolate D32 | GBMNE75872-22 | BOLD:AEZ5707 | OP050241 |
| *Haemaphysalis chordeilis* | Canada | MN991269 | GBMNC73414-20 | BOLD:AEF9661 | MN991269 |
| *Haemaphysalis chordeilis* | Canada | isolate HCTG3 |  |  | OP787254 |
| *Haemaphysalis cuspidata* | India | S7N2 |  |  | PQ410423* |
| *Haemaphysalis cuspidata* | India | S7N3 |  |  | PQ410424* |
| *Haemaphysalis cuspidata* | India | S7N1 |  |  | PQ410425* |
| *Haemaphysalis danieli* | China | Isolate Z14 |  |  | OM368292 |
| *Haemaphysalis danieli* | Pakistan | Isolate cox_1 |  |  | OP435801 |
| *Haemaphysalis doenitzi* | China |  | GBCH7606-13 | BOLD:ACH7918 | JQ346688 |
| *Haemaphysalis elliptica* | South Africa | TIC8 |  |  | PP835210 |
| *Haemaphysalis* | Thailand | TMTK002-004 | ENTJR395-08 | BOLD:AAI1967 |  |
| *Haemaphysalis* | Thailand | TMTK002-008 | ENTJR399-08 | BOLD:AAI1967 |  |
| *Haemaphysalis erinacei* | Turkey | Isolate Tokat | GBMIN116815-17 | BOLD:ADK3574 | KX901844 |
| *Haemaphysalis flava* | China | Isolate 3513 | GACAC1315-12 | BOLD:AAI6530 | JQ625688 |
| *Haemaphysalis formosensis* | Japan |  | GBMNA12171-19 | BOLD:ADC9052 | JX573135 |
| *Haemaphysalis hoodi* | Cameroon | HhFemCam | GBMNE73707-22 | BOLD:ACQ7237 | ON191014 |
| *Haemaphysalis humerosa* | Australia |  | GBCH0069-06 | BOLD:AAY1813 | AF132819 |
| *Haemaphysalis humerosa* | Australia |  | GBCH11569-13 | BOLD:ACH9091 | JX573138 |
| *Haemaphysalis hystricis* | China |  | GACAC3852-19 | BOLD:AAH6688 | MH510034 |
| *Haemaphysalis hystricis* | Japan |  | GBCH11570-13 | BOLD:AAH6688 | JX573137 |
| *Haemaphysalis hystricis* | China | Isolate A1 *H. hystricis* | GBMNE25063-21 | BOLD:AAH6688 | MZ853181 |
| *Haemaphysalis indica* | Pakistan | Isolate COX |  |  | OM480648 |
| *Haemaphysalis intermedia* | India | Isolate ICMR-VCRC GIS-WDA1001 |  |  | PQ211036 |
| *Haemaphysalis japonica* | China |  | GACAC3856-19 | BOLD:ADW4604 | NC_037246 |
| *Haemaphysalis japonica* | India | Isolate NIVEDI_2019_PK18 | GACO5383-19 | BOLD:ADG5534 | MK863383 |
| *Haemaphysalis juxtakochi* | Panama | CAS-T051 | TICKS013-12 | BOLD:ABY3817 | KF200081 |
| *Haemaphysalis juxtakochi* | Panama | CAS-T052 | TICKS014-12 | BOLD:ABY3818 | KF200120 |
| *Haemaphysalis kashmirensis* | Pakistan | Isolate HK45 |  |  | OQ096625 |
| *Haemaphysalis kinneari* | India | S10N1 |  |  | PQ410421* |
| *Haemaphysalis kinneari* | India | S10N2 |  |  | PQ410422* |
| *Haemaphysalis kopetdaghica* | Turkey | B-1115 | IBX004-20 | BOLD:AEC7082 | MT308585 |
| *Haemaphysalis* near *kyasanurensis* | India | S5AF |  |  | PQ436027* |
| *Haemaphysalis* near *kyasanurensis* | India | S5N1 |  |  | PQ436028* |
| *Haemaphysalis* near *kyasanurensis* | India | S5N2 |  |  | PQ436029* |
| *Haemaphysalis lagostrophi* | Australia | Isolate E1LaF |  |  | MN686569 |
| *Haemaphysalis leachi* | Republic of Chad | MARK45 | GBMNC73335-20 | BOLD:AEG9405 | MN663156 |
| *Haemaphysalis leachi* | Nigeria | Isolate 508 | GBMND45972-21 | BOLD:AEG9405 | MW558149 |
| *Haemaphysalis lemuris* | Madagascar | Isolate 23 |  |  | JX470177 |
| *Haemaphysalis leporispalustris* | Mexico | MX_18A | GBMNC73337-20 | BOLD:AEH0070 | MN663154 |
| *Haemaphysalis leporispalustris* | Canada | BIOUG30582-B10 | MSSCA1755-16 | BOLD:ACL3894 | MN351708 |
| *Haemaphysalis longicornis* | China | Isolate 5024 | GACAC1320-12 | BOLD:AAY1812 | JQ625693 |
| *Haemaphysalis longicornis* | Australia |  | GBCH0070-06 | BOLD:AAY1812 | AF132820 |
| *Haemaphysalis mageshimaensis* | China | Isolate Q2 |  |  | OM368289 |
| *Haemaphysalis mageshimaensis* | Japan | Isolate YY-mage09 |  |  | PQ787788 |
| *Haemaphysalis megaspinosa* | South Korea | Isolate JJRDT278/19 | GBMNE74579-22 | BOLD:ADW4604 | ON619610 |
| *Haemaphysalis minuta* | India | Isolate Kottiyoor isolate, Kannur |  |  | PQ324273 |
| *Haemaphysalis montgomeryi* | China | Isolate Yunnan |  |  | NC_058312 |
| *Haemaphysalis montgomeryi* | Pakistan | Isolate COX |  |  | OM469326 |
| *Haemaphysalis muhsamae* | Ghana | GHDO2_2020 |  |  | PV172609 |
| *Haemaphysalis muhsamae* | Republic of the Congo | MT646137 | GBMNC71128-20 | BOLD:ADZ7643 | MT646137 |
| *Haemaphysalis nadchatrami* | Malaysia | Isolate HNf2 |  |  | PP108268 |
| *Haemaphysalis nepalensis* | China |  |  |  | NC_064124 |
| *Haemaphysalis novaeguineae* | Australia | B5081b |  |  | OR350533 |
| *Haemaphysalis parva* | Turkey | Isolate Hpa-1799 | HAPA015-24 | BOLD:ADA9786 | PQ725641 |
| *Haemaphysalis pentalagi* | Japan | 3231 |  |  | MT371805 |
| *Haemaphysalis punctata* | Romania | bb.f.147.1 | GBCH11613-13 | BOLD:ABX1743 | JX394184 |
| *Haemaphysalis punctata* | United Kingdom | CCDB-04692-F07 | HPATK067-12 | BOLD:ABX1743 | PP048548 |
| *Haemaphysalis qinghaiensis* | China | Isolate LT17 | GACAC3928-19 | BOLD:ACQ4980 | MF981043 |
| *Haemaphysalis qinghaiensis* | China | Isolate Tianshui | GBA20350-14 | BOLD:ACQ4980 | JQ737094 |
| *Haemaphysalis semermis* | Malaysia | HSM1 |  |  | PQ288529 |
| *Haemaphysalis shimoga* | India | P9N1 |  |  | PQ436521* |
| *Haemaphysalis shimoga* | India | S9N2 |  |  | PQ436522* |
| *Haemaphysalis shimoga* | Cambodia | Isolate KCH2 |  |  | PQ838781 |
| *Haemaphysalis spinigera* | India | Isolate ICMR-VCRC GIS-WDA-514 |  |  | PQ213466 |
| *Haemaphysalis spinigera* | India | K1N2 |  |  | PQ432788* |
| *Haemaphysalis spinigera* | India | K1N3 |  |  | PQ432789* |
| *Haemaphysalis spinigera* | India | S1N1 |  |  | PQ432790* |
| *Haemaphysalis spinigera* | India | S1N2 |  |  | PQ432791* |
| *Haemaphysalis sulcata* | Iran | s45 | GACO3910-19 | BOLD:ACH8185 | MH532303 |
| *Haemaphysalis sulcata* | Romania | bb.f.71.3 | GBCH11608-13 | BOLD:ACH8185 | JX394189 |
| *Haemaphysalis sulcata* | Pakistan | Isolate COX1-355-Sheep | GBMNC64208-20 | BOLD:AEF9858 | MT800321 |
| *Haemaphysalis taiwana* | China |  |  |  | NC_085275 |
| *Haemaphysalis tibetensis* | China | Isolate Chayu-29 | GBMNE74870-22 | BOLD:AEX4287 | ON783071 |
| *Haemaphysalis turturis* | India | Isolate Mallampara isolate, Kasargod |  |  | PQ321229 |
| *Haemaphysalis turturis* | India | K2N1 |  |  | PQ451931* |
| *Haemaphysalis turturis* | India | K2N2 |  |  | PQ451932* |
| *Haemaphysalis turturis* | India | P2N3 |  |  | PQ451933* |
| *Haemaphysalis turturis* | India | S2N2 |  |  | PQ451934* |
| *Haemaphysalis verticalis* | China | Isolate 18-1 |  |  | KR108849 |
| *Haemaphysalis warburtoni* | China |  |  |  | NC084204 |
| *Haemaphysalis wellingtoni* | India | K8N1 |  |  | PQ444036* |
| *Haemaphysalis wellingtoni* | India | K8N2 |  |  | PQ444037* |
| *Haemaphysalis wellingtoni* | India | P8AF |  |  | PQ444038* |
| *Haemaphysalis wellingtoni* | India | P8AM |  |  | PQ444039* |
| *Haemaphysalis wellingtoni* | India | P8N1 |  |  | PQ444040* |
| *Haemaphysalis wellingtoni* | Singapore | F34-11-24-M |  |  | PV242077 |
| *Haemaphysalis yeni* | Japan | 3599 |  |  | OL741745 |
| *Alloceraea colasbelcouri* | China | isolate Y1 |  |  | OM368290 |
| *Alloceraea inermis* | Iran | i43 | GACO3229-19 | BOLD:ACH7916 | MH532295 |
| *Alloceraea kitaokai* | Turkey | Isolate QDN-69 | GBMNE75906-22 | BOLD:AEY6859 | OP107278 |
| *Alloceraea kolonini* | China |  |  |  | MZ054209 |
