# Supplementary material for: Integrative taxonomy of Haemaphysalis (Acari: Ixodidae) from the Western Ghats, India: Morphological and molecular characterization and implications
Source: PLoS One. 2026 May 7;21(5):e0348592. doi: 10.1371/journal.pone.0348592 (PMC13152140; doi:10.1371/journal.pone.0348592)
Supplement: S2 Table — Their provenances, BOLD information and GenBank accession numbers. Newly generated sequences are marked with asterisk. (DOCX) [file pone.0348592.s002.docx]

**S2 Table.** *Haemaphysalis* (*Kaiseriana*) *bispinosa* sequences used for haplotype and population study. Their provenances, BOLD information and GenBank accession numbers. Newly generated sequences are marked with asterisk.

| **Species** | **Provenance** | **Voucher code** | **BOLD:Process id** | **BOLD:BIN** | **GenBank Accession no.** |
| --- | --- | --- | --- | --- | --- |
| *Haemaphysalis bispinosa* | India | K3N2 |  |  | PQ432748* |
| *Haemaphysalis bispinosa* | India | S3N1 |  |  | PQ432749* |
| *Haemaphysalis bispinosa* | India | isolate Wayanad 1 |  |  | OM442981 |
| *Haemaphysalis bispinosa* | India | isolate NIVEDI_2019_PK38 |  |  | MN106409 |
| *Haemaphysalis bispinosa* | India | KFD-S-551_L2 |  |  | PQ687485 |
| *Haemaphysalis bispinosa* | India | isolate Ollur |  |  | PV798834 |
| *Haemaphysalis bispinosa* | India | KFD-S-2899_LP10 |  |  | PQ687492 |
| *Haemaphysalis bispinosa* | India | isolate NIVEDI_2022_PK59 |  |  | ON413877 |
| *Haemaphysalis bispinosa* | India | isolate NIVEDI_2022_PK58 |  |  | ON413860 |
| *Haemaphysalis bispinosa* | India | isolate NIVEDI_2022_PK56 |  |  | ON413859 |
| *Haemaphysalis bispinosa* | India | isolate MU790 |  |  | PV739674 |
| *Haemaphysalis bispinosa* | India | isolate Ollur |  |  | PV746300 |
| *Haemaphysalis bispinosa* | India | isolate NIVEDI_2019_PK30 | GACO5389-19 | BOLD: ADV1020 | MK863389 |
| *Haemaphysalis bispinosa* | India | isolate NIVEDI_2019_PK34 | GACO5398-19 | BOLD: ADV1020 | MK952745 |
| *Haemaphysalis bispinosa* | India | isolate NIVEDI_2022_PK62 |  |  | ON416654 |
| *Haemaphysalis bispinosa* | India | isolate NIVEDI_2022_PK50 |  |  | ON413787 |
| *Haemaphysalis bispinosa* | India | isolate NIVEDI_2019_PK08 | GACO5367-19 | BOLD: ADV1020 | MK820028 |
| *Haemaphysalis bispinosa* | India | isolate NIVEDI_2019_PK20 | GACO5385-19 | BOLD: ADV1020 | MK863385 |
| *Haemaphysalis bispinosa* | India | isolate NIVEDI_2019_PK31 | GACO5392-19 | BOLD: ADV1020 | MK889215 |
| *Haemaphysalis bispinosa* | India | isolate NIVEDI_2019_PK24 | GACO5391-19 | BOLD: ADV1020 | MK889214 |
| *Haemaphysalis bispinosa* | India | isolate NIVEDI_2019_PK33 | GACO5394-19 | BOLD: ADV1020 | MK889217 |
| *Haemaphysalis bispinosa* | India | isolate Thrissur |  |  | PV639340 |
| *Haemaphysalis bispinosa* | India | isolate NIVEDI_2019_PK09 | GACO5368-19 | BOLD: ADV1020 | MK820029 |
| *Haemaphysalis bispinosa* | India | isolate NIVEDI_2019_PK10 | GACO5369-19 | BOLD: ADV1020 | MK820030 |
| *Haemaphysalis bispinosa* | Malaysia | UPMBTU_T02 |  |  | OR742949 |
| *Haemaphysalis bispinosa* | Malaysia | UPMBTU_T07 |  |  | OR742954 |
| *Haemaphysalis bispinosa* | Pakistan | isolate LRK-C23 |  |  | PV687000 |
| *Haemaphysalis bispinosa* | Pakistan | isolate Thar-G25 |  |  | PV687003 |
| *Haemaphysalis bispinosa* | Pakistan | isolate LRK-B21 |  |  | PV686999 |
| *Haemaphysalis bispinosa* | Pakistan | isolate HYD-G31 |  |  | PV687002 |
| *Haemaphysalis bispinosa* | Pakistan | isolate LRK-G55 |  |  | PV687004 |
| *Haemaphysalis bispinosa* | Pakistan | isolate LRK-G21 |  |  | PV687001 |
| *Haemaphysalis bispinosa* | Pakistan | isolate HYD-G9 |  |  | PV686998 |
| *Haemaphysalis bispinosa* | Pakistan | PAK204 |  |  | PV594384 |
| *Haemaphysalis bispinosa* | Pakistan | isolate COX |  |  | ON564620 |
| *Haemaphysalis bispinosa* | Pakistan | isolate HB1 |  |  | OQ860248 |
| *Haemaphysalis bispinosa* | Pakistan | PK01 |  |  | PP325825 |
| *Haemaphysalis bispinosa* | Bangladesh | TK0H | GACO4037-19 | BOLD: ADV1020 | MK202801 |
| *Haemaphysalis bispinosa* | Bangladesh | B2 | GACO3119-19 | BOLD: ADV1020 | MK140595 |
| *Haemaphysalis bispinosa* | Bangladesh |  | GACO5061-19 | BOLD: ADV1020 | MK089788 |
| *Haemaphysalis bispinosa* | Bangladesh | TK0G | GACO4036-19 | BOLD: ADV1020 | MK188368 |
| *Haemaphysalis bispinosa* | Bangladesh | TK0T2 | GACO4042-19 | BOLD: ADV1020 | MK318817 |
| *Haemaphysalis bispinosa* | Bangladesh | TK0O5 | GACO4038-19 | BOLD: ADV1020 | MK240315 |
| *Haemaphysalis bispinosa* | Bangladesh | TK0R2 | GACO4040-19 | BOLD: ADV1020 | MK269314 |
| *Haemaphysalis bispinosa* | Bangladesh | TKOI2 | GACO4043-19 | BOLD: ADV1020 | MK234913 |
| *Haemaphysalis bispinosa* | Bangladesh | TK0T | GACO4041-19 | BOLD: ADV1020 | MK224466 |
| *Haemaphysalis bispinosa* | Bangladesh | TK0Q5 | GACO4039-19 | BOLD: ADV1020 | MK256941 |
| *Haemaphysalis bispinosa* | China |  |  |  | OP383037 |
| *Haemaphysalis bispinosa* | China |  |  |  | NC_071765 |
| *Haemaphysalis bispinosa* | Tanzania | isolate COX1_S05_Tz |  |  | PV478291 |
| *Haemaphysalis bispinosa* | Tanzania | isolate COX1_S04_Tz |  |  | PV478290 |
| *Haemaphysalis bispinosa* | Thailand | H02_NE_Np |  |  | OR335053 |
| *Haemaphysalis bispinosa* | Thailand | H08_NE_Sn |  |  | OR335059 |
| *Haemaphysalis bispinosa* | Thailand | H10_NE_Sn |  |  | OR335061 |
| *Haemaphysalis bispinosa* | Thailand | HbCOI_NPM_27 |  |  | OM760850 |
| *Haemaphysalis bispinosa* | Thailand | isolate HbCOI_NPM_13 |  |  | OM760846 |
| *Haemaphysalis bispinosa* | Thailand | isolate HbCOI_NPM_14 |  |  | OM760847 |
| *Haemaphysalis bispinosa* | Thailand | isolate HbCOI_NPM_15 |  |  | OM760848 |
| *Haemaphysalis bispinosa* | Thailand | isolate HbCOI_NPM_18 |  |  | OM760849 |
| *Haemaphysalis bispinosa* | Thailand | isolate HbCOI_NPM_45 |  |  | OM760851 |
| *Haemaphysalis bispinosa* | Thailand | isolate HbCOI_UDN_58 |  |  | OM760852 |
| *Haemaphysalis bispinosa* | Thailand | isolate HbCOI_SNK_63 |  |  | OM760853 |
| *Haemaphysalis bispinosa* | Thailand | isolate HbCOI_BNK_93 |  |  | OM760854 |
| *Haemaphysalis bispinosa* | Thailand | isolate HbCOI_RET_99 |  |  | OM760855 |
| *Haemaphysalis bispinosa* | Thailand | H01_NE_Np |  |  | OR335052 |
| *Haemaphysalis bispinosa* | Thailand | H03_NE_Sn |  |  | OR335054 |
| *Haemaphysalis bispinosa* | Thailand | H04_NE_Sn |  |  | OR335055 |
| *Haemaphysalis bispinosa* | Thailand | H05_NE_Sn |  |  | OR335056 |
| *Haemaphysalis bispinosa* | Thailand | H06_NE_Sn |  |  | OR335057 |
| *Haemaphysalis bispinosa* | Thailand | H07_NE_Sn |  |  | OR335058 |
| *Haemaphysalis bispinosa* | Thailand | H09_NE_Sn |  |  | OR335060 |
| *Haemaphysalis bispinosa* | Thailand | H11_NE_Yo |  |  | OR335062 |
| *Haemaphysalis bispinosa* | Thailand | H12_NE_Ur |  |  | OR335063 |
| *Haemaphysalis bispinosa* | Java | isolate C2Ampel |  |  | ON778582 |
| *Haemaphysalis bispinosa* | Java | isolate C8Nogosari |  |  | ON778584 |
| *Haemaphysalis bispinosa* | Java | isolate CDMusuk |  |  | ON778586 |
| *Haemaphysalis bispinosa* | Java | isolate CGTeras |  |  | ON778587 |
| *Haemaphysalis bispinosa* | Java | isolate CHMojosongo |  |  | ON778588 |
| *Haemaphysalis bispinosa* | Java | isolate C1Musuk |  |  | ON778581 |
| *Haemaphysalis bispinosa* | Java | isolate C7Mojosongo |  |  | ON778583 |
| *Haemaphysalis bispinosa* | Vietnam | isolate VQ9 |  |  | PQ439198 |
| *Haemaphysalis bispinosa* | Singapore | M71-10-22-N |  |  | PV242080 |
