# Supplementary figures and images for: Integrative taxonomy of Haemaphysalis (Acari: Ixodidae) from the Western Ghats, India: Morphological and molecular characterization and implications
Source: PLoS One. 2026 May 7;21(5):e0348592. doi: 10.1371/journal.pone.0348592 (PMC13152140; doi:10.1371/journal.pone.0348592)

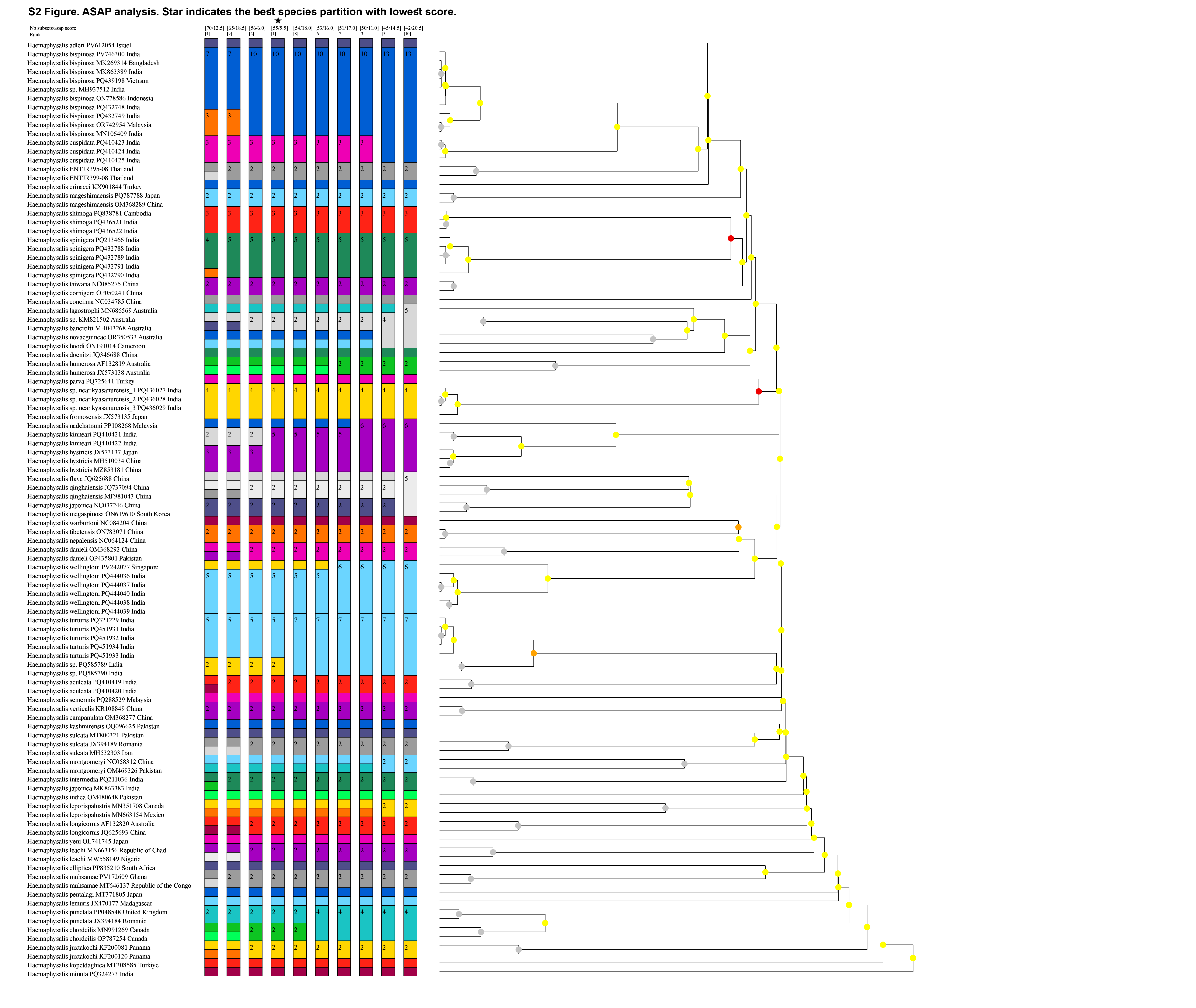

Supplement: S2 Fig — The left panel shows partitioning results across different ASAP scores, with each color representing a putative species hypothesis. The right panel depicts the corresponding phylogenetic tree, where yellow and red circles indicate node support values. (TIF) [file pone.0348592.s004.tif]

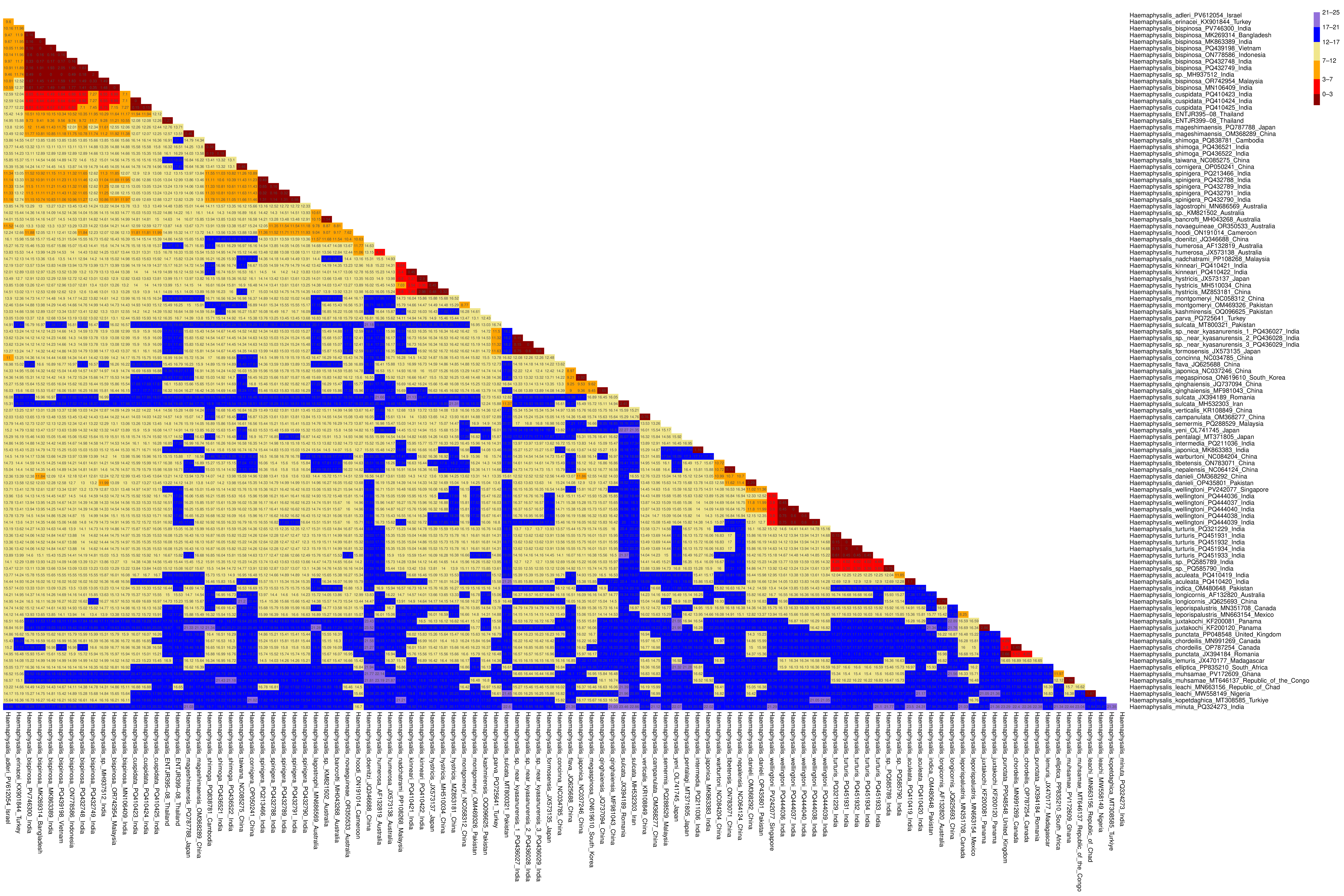

Supplement: S3 Fig — The matrix illustrates uncorrected p-distances ranging from 0% (blue, genetically identical or very closely related) to 25% (dark red, highly divergent). Each row and column corresponds to a sequence labelled with species name, GenBank accession number, and country of origin. (TIF) [file pone.0348592.s005.TIF]
