# Supplementary material for: Integrative taxonomy of Haemaphysalis (Acari: Ixodidae) from the Western Ghats, India: Morphological and molecular characterization and implications
Source: PLoS One. 2026 May 7;21(5):e0348592. doi: 10.1371/journal.pone.0348592 (PMC13152140; doi:10.1371/journal.pone.0348592)
Supplement: Attachment — Submitted filename: Response to Reviewers.docx [file pone.0348592.s006.docx]

**Point-by-Point Response to Editor’s Comments**

We sincerely thank the Editor for the constructive suggestions that have helped improve the clarity and quality of our manuscript. Below we provide our point-by-point responses.

- I would suggest modifying the title again as follows: “…morphological and molecular characterization and implications.”

Response:

Thank you for the suggestion. The title has been revised accordingly to include the phrase “morphological and molecular characterization and implications.”

- Line 24: change to: “were confirmed by COI sequence analyses.”

Response:

The sentence has been revised as suggested.

- Line 37: change to: “COI for *Haemaphysalis* identification at the species level.”

Response:

The sentence has been modified accordingly.

- Line 128: change to: “Molecular procedures.”

Response:

The subheading has been revised from the previous wording to “Molecular procedures,” as suggested.

- Line 209: I would suggest deleting the subheading and merging this section with the previous one into a single paragraph.

Response:

As recommended, the subheading has been removed and the text has been merged with the preceding section to form a single coherent paragraph.

- Line 235: The authority of each species should be indicated when mentioned for the first time.

Response:

Thank you for pointing this out. The taxonomic authorities for each species have now been included at their first mention in the manuscript.

- Line 240: It is not clear whether “the most abundant species” refers to adults or nymphs; please specify.

Response:

The sentence has been revised to explicitly indicate whether the statement refers to adults or nymphs.

- Line 384: I suggest indicating the percentage of identity, even if low. Therefore, change to: “*Haemaphysalis* (*Kaiseriana*) *aculeata* and *H.* (*Kaiseriana*) *cuspidata* showed an XX% similarity with YYY, since reference COI sequences were …”

Response:

Thank you for the suggestion. The sentence has been revised to include the percentage identity values. It now specifies the sequence similarity observed between *Haemaphysalis* (*Kaiseriana*) *aculeata* and *H.* (*Kaiseriana*) *cuspidata* and the closest available reference sequences.

- Line 387: This paragraph needs to be more concise.

Response: The paragraph has been edited for conciseness, removing redundant statements and improving clarity while retaining the essential information.

- Lines 439 and 446: Please indicate the values for low p-distance and high divergence.

Response:

The manuscript has been revised to explicitly include the numerical values corresponding to low p-distance and high divergence in the relevant sentences.

- Line 455: I suggest including data described in this paragraph in a table and summarizing it.

Response:

We appreciate this helpful suggestion. The data previously described in the paragraph have now been organized into a new table to improve clarity and readability. The text has been shortened accordingly to provide a brief summary referring to the table.
